# Numerical Solution of Blood Flow and Mass Transport in an Elastic Tube with Multiple Stenoses

**DOI:** 10.1155/2020/7609562

**Published:** 2020-01-31

**Authors:** Reima D. Alsemiry, Sarifuddin, Prashanta K. Mandal, Hamed M. Sayed, Norsarahaida Amin

**Affiliations:** ^1^Department of Mathematics, Faculty of Science, Taibah University, P.O. Box 89, Yanbu 41911, Saudi Arabia; ^2^Department of Mathematical Sciences, Universiti Teknologi Malaysia, 81310 UTM, Johor Bahru, Johor, Malaysia; ^3^Department of Mathematics, Berhampore College, Baharampur 742101, West Bengal, India; ^4^Department of Mathematics, Visva-Bharati University, Santiniketan 731235, West Bengal, India; ^5^Department of Mathematics, Faculty of Education, Ain Shams University, Roxy 11757, Cairo, Egypt

## Abstract

The simultaneous effect of flexible wall and multiple stenoses on the flow and mass transfer of blood is investigated through numerical computation and simulations. The solution is obtained using the Marker and Cell technique on an axisymmetric model of Newtonian blood flow. The results compare favorably with physical observations where the pulsatile boundary condition and double stenoses result in a higher pressure drop across the stenoses. The streamlines, the iso-concentration lines, the Sherwood number, and the mass concentration variations along the entire wall segment provide a comprehensive analysis of the mass transport characteristics. The double stenoses and pulsatile inlet conditions increase the number of recirculation regions and effect a higher mass transfer rate at the throat, whereby more mass is expected to accumulate and cause further stenosis.

## 1. Introduction

Caro et al. [1] postulated that atherosclerosis, which is a narrowing of the artery as a result of plaque build-up may occur due to shear-dependent mass transfer mechanism between blood cholesterol and the arterial wall. Cholesterol exists in blood in the form of low density lipoproteins (LDLs) whose deposition along the walls of the artery is a key step in atherogenesis, which would lead to stenosis. Stenosis can affect the velocity of blood flowing through the artery, affecting blood pressure, collapsing the heart, which could in turn lead to disastrous consequences. Thus, an understanding of the behavior of local mass transport in arterial stenosis is important in the study of the formation and development of atherosclerotic lesions for appropriate assessment on the possible correlation between the site of atherosclerotic lesions and the pattern of mass transport.

Ethier [2] carried out computational modelling of mass transfer and studied its links to atherosclerosis. Other studies on mass transport and fluid flow in stenotic arteries of axisymmetric and asymmetric models have been carried out by [3–6]. In these studies, the arterial wall was considered as rigid and the artery is assumed to have single mild stenosis, in which the geometry of the stenosis is represented by the usual cosine curve along with a restriction that the ratio of the severity of stenosis and the radius of the artery is very small. In reality, this is not the case where in many medical situations, the patient is found to have multiple stenoses in the same arterial segment.

Investigations on the effect of multiple stenoses on blood flow have been carried out amongst others by [7–10]. These studies showed that from both experimental results and theoretical calculations, the total effect of a series of noncritical stenoses is approximately equal to the sum of their individual effects where they can be critical and produce symptoms of arterial insufficiency. The flow energy loss due to the presence of the stenoses, which is directly related to the pressure drop across them, increases with the number of stenoses but is not strongly dependent on the spacing between them. The authors of [11–19] have also investigated blood flow through multiple stenoses; however, these studies have not considered the mass transfer.

Another aspect to be considered in arterial blood flow is the cyclic nature of the heart pump which creates pulsatile conditions in the arteries, giving rise to unsteady flow. It is observed that most CFD models of arterial hemodynamics make the simplifying assumptions of rigid walls and fully developed inlet velocities (cf. [13–19]). But the arteries are not rigid tubes. They adapt to varying flow conditions by enlarging or shrinking. All of these physiological conditions make the modelling and consequently the solution to be almost impossible to be obtained analytically and challenging computationally. Nandakumar and Anand [20] studied steady and pulsatile flow of blood through a channel with single as well as double stenoses on the assumption that the pulsations of flow are damped in the small vessels; thus the flow is effectively steady in the capillaries and the veins while Liu and Tang [21] investigated the influence of distal stenosis on blood flow through curved arteries with two stenoses. But again, these studies on pulsatile flow have also not considered the mass transfer. In another study, Layek et al. [22] investigated the effect of multiple stenoses on the flow of Newtonian fluid in a rigid tube and opined that the disturbance created by the constrictions is mainly concentrated at the downstream of the last constriction. Considering the flow of Newtonian fluid in a two-dimensional channel having a single constriction, Layek and Midya [23] concluded that the maximum stress and the length of the recirculation region associated with two shear layers of the constriction do increase with the increasing area reduction of the constriction. They further concluded that the flow-field separates after the symmetry breaking bifurcation, and the symmetry of the flow depends on Reynolds' number and the height of the constriction. The flow of a fluid having hematocrit-dependent viscosity past a tube with partially overlapped constriction has been investigated by Layek et al. [24]. They observed that the peak value of the wall shear stress decreases with increasing haematocrit parameter while a reverse trend is observed for the flow separation region. They also opined that the deformability of the wall does reduce the wall shear stress as compared to the rigid wall case. All these studies [22–24] ignored the flow pulsatility and/or consideration of multiple constrictions, and the mass transfer as well which plays a pivotal role in the genesis and evolution of atherosclerosis.

Based on the gap established above, with regard to studies involving mass transfer, the following work seeks to analyze the flow and mass transfer characteristics of pulsatile blood flow through an artery with double stenoses. The fluid considered is Newtonian in an axisymmetric setting, while the pair of stenoses vary in severities, lengths, and distances between them. The equation for stenoses is given in an algebraic form which could represent both moderate and severe stenoses instead of the usual cosine function which could only describe mild stenosis. The objective of the present study lies in the consideration of the transport of mass as well as momentum together through a tube with a flexible wall, resembling the flexibility of the artery in the presence of double stenoses. The flow pulsatility cannot be ruled out from the present investigation.

## 2. Formulation of the Problem

We consider a fully developed two-dimensional axisymmetric flow of an incompressible Newtonian fluid of density *ρ* in a tube. The relevant equations of motions in vector forms are the continuity, momentum, and mass as follows:

(1) $$\begin{array}{c} \nabla \cdot V=0, \end{array}$$

(2) $$\begin{array}{c} \rho \frac{\text{D}V}{\text{D}t}=-\nabla P+\mu \nabla^{2}V, \end{array}$$

(3) $$\begin{array}{c} \frac{\text{D}C}{\text{D}t}=D_{\text{m}}\nabla^{2}C, \end{array}$$

with D/D*t* is the material derivative, **V**=(*u*, 0, *w*) where *u* and *w* are the radial and axial velocity components, respectively, *p* is the pressure, *μ* is the constant viscosity, *C* is the mass concentration, and *D*_m_ is the coefficient of mass diffusion.

In the cylindrical coordinate system, the corresponding equations (1)–(3) are written in a conservative form as follows:

(4) $$\begin{array}{c} \frac{1}{r}\frac{\partial }{\partial r}\left(ru\right)+\frac{\partial w}{\partial z}=0, \end{array}$$

(5) $$\begin{array}{c} \rho \left[\frac{\partial w}{\partial t}+\frac{\partial \left(wu\right)}{\partial r}+\frac{\partial w^{2}}{\partial z}+\frac{wu}{r}\right]=-\frac{\partial p}{\partial z}+\mu \left[\frac{1}{r}\frac{\partial }{\partial r}\left(r\frac{\partial w}{\partial r}\right)+\frac{\partial^{2}w}{\partial z^{2}}\right], \end{array}$$

(6) $$\begin{array}{c} \rho \left[\frac{\partial u}{\partial t}+\frac{\partial \left(wu\right)}{\partial z}+\frac{\partial u^{2}}{\partial r}+\frac{u^{2}}{r}\right]=-\frac{\partial p}{\partial r}+\mu \left[\frac{1}{r}\frac{\partial }{\partial r}\left(r\frac{\partial u}{\partial r}\right)-\frac{u}{r^{2}}+\frac{\partial^{2}u}{\partial z^{2}}\right], \end{array}$$

(7) $$\begin{array}{c} \frac{\partial C}{\partial t}+u\frac{\partial C}{\partial r}+w\frac{\partial C}{\partial z}=D_{\text{m}}\left[\frac{\partial^{2}C}{\partial z^{2}}+\frac{1}{r}\frac{\partial C}{\partial r}+\frac{\partial^{2}C}{\partial r^{2}}\right]. \end{array}$$

**Figure 1:**
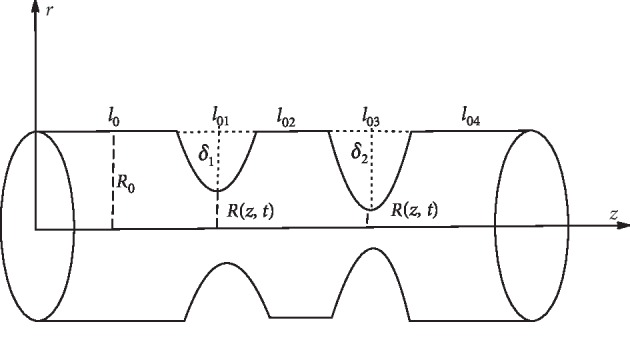
Schematic diagram for the double arterial stenoses.

The schematic diagram for the double stenoses is given in Figure 1, where *r*=*R*(*z*, *t*) is the radius of the artery in the stenotic region and *R*_0_ is the radius of the artery in the nonstenotic regions. *δ*_1_, *δ*_2_ are the critical heights of the first and second stenosis respectively; *l*_0_ is the inlet segment, *l*_02_ is the distance between stenoses, *l*_01_, *l*_03_ are the lengths of stenoses, and *L* is the length of the arterial segment under consideration.

The equations describing the stenoses are given by the following:

(8) $$\begin{array}{c} R\left(z,t\right)=\left\{\begin{array}{cc} R_{0}a_{1}\left(t\right), & 0\leq z\leq l_{0},\\ \left(R_{0}+\frac{4\delta_{1}}{l_{01}^{2}}\left[{\left(z-l_{01}\right)}^{2}-l_{01}\left(z-l_{01}\right)\right]\right)a_{1}\left(t\right),\; & l_{0}\leq z\leq l_{0}+l_{01},\\ R_{0}a_{1}\left(t\right), & l_{0}+l_{01}\leq z\leq l_{0}+l_{01}+l_{02},\\ \left(R_{0}+\frac{4\delta_{2}}{l_{02}^{2}}\left[{\left(z-d\right)}^{2}-l_{02}\left(z-d\right)\right]\right)a_{1}\left(t\right),\; & l_{0}+l_{01}+l_{02}\leq z\leq l_{0}+l_{01}+l_{02}+l_{03},\\ R_{0}a_{1}\left(t\right),\; & l_{0}+l_{01}+l_{02}+l_{03}\leq z\leq L. \end{array}\right. \end{array}$$

The time-variant parameter *a*_1_(*t*) is given by *a*_1_(*t*)=1+*k* cos(*ωt*) with *k* representing the amplitude parameter and *ω* the angular frequency is given by *ω*=2*πf*_*p*_, *f*_*p*_ being the pulse frequency and *d*=*l*_0_+*l*_01_+*l*_02_. To the best of our knowledge equation (8) is the first equation to address double stenoses without any control on the severity of stenoses which has not been considered before.

### 2.1. Boundary Conditions

(9) $$\begin{array}{c} \text{At }r=0:u\left(r,z,t\right)=0,\;\frac{\partial w\left(r,z,t\right)}{\partial r}=0,\frac{\partial C\left(r,z,t\right)}{\partial r}=0, \end{array}$$

(10) $$\begin{array}{c} \text{At }r=R\left(z,t\right):u\left(r,z,t\right)=\frac{\partial R}{\partial t},\\ w\left(r,z,t\right)=0,C\left(r,z,t\right)=0, \end{array}$$

(11a) $$\begin{array}{c} \text{At }z=0:u\left(r,z,0\right)=0,C\left(r,z,0\right)=C_{s}, \end{array}$$

(11b) $$\begin{array}{c} w\left(r,z,0\right)=\left\{\begin{array}{c} U\left[1-{\left(\frac{r}{R}\right)}^{2}\right]\left(\text{parabolic inlet}\right),\\ U\left[1-{\left(\frac{r}{R}\right)}^{2}\right]\left[1+k\;\cos\left(\omega t\right)\right]\left(\text{pulsatile inlet}\right), \end{array}\right.\; \end{array}$$

where *U* is the cross-sectional average velocity of the fluid and *C*_*s*_ is a constant.

(12) $$\begin{array}{c} \text{At }z=L:\frac{\partial u\left(r,z,t\right)}{\partial z}=0,\;\frac{\partial w\left(r,z,t\right)}{\partial z}=0,\frac{\partial C\left(r,z,t\right)}{\partial z}=0. \end{array}$$

## 3. Solution Procedure

The solution procedure involves the nondimensionalization, radial coordinate transformation, and the finite-difference Marker and Cell method (MAC) initially proposed by Harlow and Welch [25]. Sarifuddin et al. [26, 27] and Mustapha et al. [15, 16] have used the method to solve blood flow problems.

### 3.1. Nondimensionalization of the Equations

  The nondimensional variables and parameters introduced are as follows:

(13) $$\begin{array}{c} \bar{r}=\frac{r}{R_{0}},\;\bar{z}=\frac{z}{R_{0}},\;\bar{u}=\frac{u}{U},\;\bar{w}=\frac{w}{U},\;\bar{t}=\frac{tU}{R_{0}},\;\bar{p}=\frac{p}{\rho U^{2}}\text{,}\\ \bar{C}=\frac{C}{C_{s}},\;{\bar{\delta }}_{1}=\frac{\delta_{1}}{R_{0}},\;{\bar{\delta }}_{2}=\frac{\delta_{2}}{R_{0}},\;{\bar{l}}_{0}=\frac{l_{0}}{R_{0}},\;{\bar{l}}_{01}=\frac{l_{01}}{R_{0}},\;{\bar{l}}_{02}=\frac{l_{02}}{R_{0}},\\ {\bar{l}}_{03}=\frac{l_{03}}{R_{0}},\;\bar{R}=\frac{R}{R_{0}}. \end{array}$$

Using (13), equations (4)–(7) have their respective nondimensional forms as follows (omitting bar):

(14) $$\begin{array}{c} \frac{\partial }{\partial r}\left(ru\right)+r\frac{\partial w}{\partial z}=0, \end{array}$$

(15) $$\begin{array}{c} \frac{\partial w}{\partial t}=-\frac{\partial \left(wu\right)}{\partial r}-\frac{\partial w^{2}}{\partial z}-\frac{wu}{r}-\frac{\partial p}{\partial z}+\frac{1}{\text{Re}}\left[\frac{1}{r}\frac{\partial }{\partial r}\left(r\frac{\partial w}{\partial r}\right)+\frac{\partial^{2}w}{\partial z^{2}}\right], \end{array}$$

(16) $$\begin{array}{c} \frac{\partial u}{\partial t}=-\frac{\partial \left(wu\right)}{\partial z}-\frac{\partial u^{2}}{\partial r}-\frac{u^{2}}{r}-\frac{\partial p}{\partial r}+\frac{1}{\text{Re}}\left[\frac{1}{r}\frac{\partial }{\partial r}\left(r\frac{\partial u}{\partial r}\right)-\frac{u}{r^{2}}+\frac{\partial^{2}u}{\partial z^{2}}\right], \end{array}$$

(17) $$\begin{array}{c} \frac{\partial C}{\partial t}+u\frac{\partial C}{\partial r}+w\frac{\partial C}{\partial z}=\frac{1}{\text{ReSc}}\left[\frac{\partial^{2}C}{\partial z^{2}}+\frac{1}{r}\frac{\partial C}{\partial r}+\frac{\partial^{2}C}{\partial r^{2}}\right]. \end{array}$$

The boundary conditions (9)–(12) reduce to their respective dimensionless forms:

(18) $$\begin{array}{c} u\left(r,z,t\right)=0,\;\frac{\partial w\left(r,z,t\right)}{\partial r}=0,\;\frac{\partial C\left(r,z,t\right)}{\partial r}=0,\text{ at }r=0, \end{array}$$

(19) $$\begin{array}{c} u\left(r,z,t\right)=\frac{\partial R}{\partial t},\;w\left(r,z,t\right)=0,\;C\left(r,z,t\right)=0,\text{ at }r=R\left(z,t\right), \end{array}$$

(20a) $$\begin{array}{c} u\left(r,z,0\right)=0,C\left(r,z,0\right)=1, \end{array}$$

(20b) wr,z,0=1−rR2,1−rR21+k cosα2Ret,at z=0,

(21) $$\begin{array}{c} \frac{\partial u\left(r,z,t\right)}{\partial z}=0,\;\frac{\partial w\left(r,z,t\right)}{\partial z}=0,\;\frac{\partial C\left(r,z,t\right)}{\partial z}=0,\text{at }z=L, \end{array}$$

where Re is the Reynolds number, Sc is the Schmidt number and *α* is the Womersley number defined as follows:

(22) Re=ρUR0μ, Sc=μρDm, α=R0ωρμ.

### 3.2. Radial Coordinate Transformation

With the introduction of a radial coordinate transformation *x*=*r*/*R*(*z*, *t*), equations (14)–(17) now become as follows:

(23) $$\begin{array}{c} xR\frac{\partial w}{\partial z}-x^{2}\frac{\partial w}{\partial x}\frac{\partial R}{\partial z}+\frac{\partial \left(xu\right)}{\partial x}=0, \end{array}$$

(24) ∂w∂t=xR∂R∂t∂w∂x−1R∂wu∂x−∂w2∂z+xR∂R∂z∂w2∂x−wuxR−∂p∂z+xR∂R∂z∂p∂x+1Re1R2+xR∂R∂z2∂2w∂x2 +1xR2+3xR2∂R∂z2−xR∂2R∂z2∂w∂x−2xR∂R∂z∂2w∂x ∂z+∂2w∂z2,

(25) ∂u∂t=xR∂R∂t∂u∂x−1R∂u2∂x−∂wu∂z+xR∂R∂z∂wu∂x−u2xR−1R∂p∂x+1Re1R2+xR∂R∂z2∂2u∂x2+1xR2+3xR2∂R∂z2−xR∂2R∂z2∂u∂x−2xR∂R∂z∂2u∂x ∂z+∂2u∂z2−ux2R2,

(26) ∂C∂t=xR∂R∂t∂C∂x−uR∂C∂x−w∂C∂z−xR∂C∂x∂R∂z+1Re·Sc1R2+xR∂R∂z2∂2C∂x2+1xR2+3xR2∂R∂z2−xR∂2R∂z2∂C∂x−2xR∂R∂z∂2C∂x ∂z+∂2C∂z2,

and the boundary conditions (18)–(21) become

(27) $$\begin{array}{c} u\left(x,z,t\right)=0,\;\frac{\partial w\left(x,z,t\right)}{\partial x}=0,\;\frac{\partial C\left(x,z,t\right)\;}{\partial x}=0,\text{ at }x=0, \end{array}$$

(28) $$\begin{array}{c} u\left(x,z,t\right)=\frac{\partial R}{\partial t},\;w\left(x,z,t\right)=0,\;C\left(x,z,t\right)=0,\text{ at }x=1,\\ \text{at }z=L. \end{array}$$

(29a) $$\begin{array}{c} u\left(x,z,0\right)=0,C\left(x,z,0\right)=1,\\ \text{at }z=L. \end{array}$$

(29b) wx,z,0=1−x2,1−x21+k cosα2Ret,at z=0,at z=L.

(30) $$\begin{array}{c} \frac{\partial u\left(x,z,t\right)}{\partial z}=0,\;\frac{\partial w\left(x,z,t\right)}{\partial z}=0,\;\frac{\partial C\left(x,z,t\right)}{\partial z}=0,\\ \text{at }z=L. \end{array}$$

### 3.3. Finite-Difference Method

**Figure 2:**
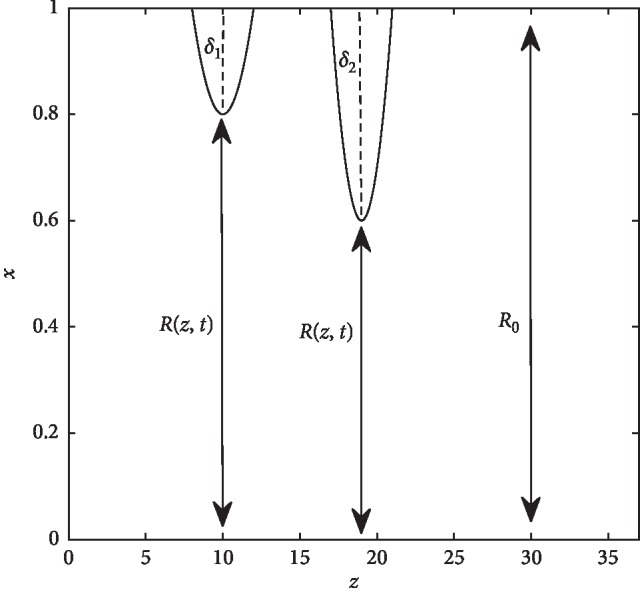
Schematic computational domain.

**Figure 3:**
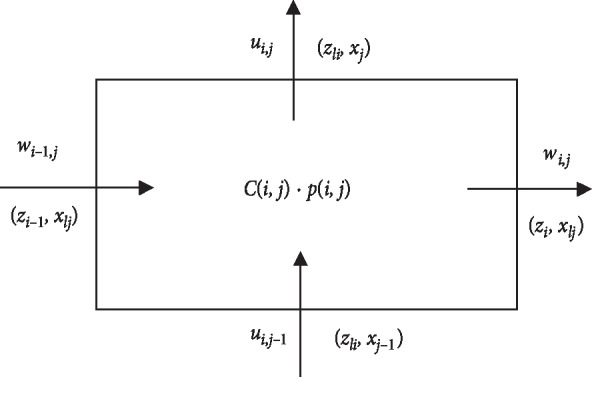
A typical MAC cell.

The solution procedure consists of discretization of the governing equations, combining the discretized forms of the momentum and continuity equations to obtain the Poisson equation for pressure, the successive overrelaxation (SOR) method, and the pressure and velocity corrections. The schematic computational domain is given in Figure 2. The velocities and pressure are calculated at different locations of the control volume, as indicated in Figure 3. The difference equations are derived at three distinct cells, each corresponding to the continuity, axial and radial momentum equations.

The discretization of the time derivative terms is based on the first-order accurate two-level forward time differencing formula, while the convective terms in the momentum equations are discretized with a hybrid formula consisting of central differencing and second-order upwinding scheme (cf. Courant et al. [28]). The diffusive terms are discretized using second-order accurate three-point central difference formula. Thus in a finite-difference formula with *x*=*j*Δ*x*,  *z*=*i*Δ*z*,  *t*=*n*Δ*t* and  *p*(*z*, *x*, *t*)=*p*(*i*Δ*z*, *j*Δ*x*, *n*Δ*t*)=*p*_*i*,*j*_^*n*^where *n* refers to time and Δ*t* is the time increment. The length and width of the (*i*, *j*)^*n*^ cell of the control volume are represented by Δ*z* and Δ*x*, respectively.

The discretized version of the continuity equation (24) at the (*i, j*) cell is

(31) $$\begin{array}{c} x_{lj}R_{li}^{n}\left(\frac{w_{i,j}^{n}-w_{i-1,j}^{n}}{\Delta z}\right)-{\left(x_{lj}\right)}^{2}\left(\frac{w_{t}-w_{b}}{\Delta x}\right){\left(\frac{\partial R}{\partial z}\right)}_{li}^{n}+\left(\frac{x_{j}u_{i,j}^{n}-x_{j-1}u_{i,j-1}^{n}}{\Delta x}\right)=0, \end{array}$$

where  *R*_*li*_=*R*(*z*_*li*_), *z*_*li*_=*z*_*i*_ − (Δ*z*/2),  *x*_*lj*_=*x*_*j*_ − (Δ*x*/2),  *w*_*t*_=0.25(*w*_*i*,*j*_^*n*^+*w*_*i*,*j*+1_^*n*^+*w*_*i*−1,*j*_^*n*^+*w*_*i*−1,*j*+1_^*n*^), *w*_*b*_=0.25(*w*_*i*,*j*_^*n*^+*w*_*i*,*j*−1_^*n*^+*w*_*i*−1,*j*_^*n*^+*w*_*i*−1,*j*−1_^*n*^).

Here (*z*_*li*_, *x*_*lj*_) and (*z*_*i*_, *x*_*j*_) represent the respective coordinates of the center of the cell and the cell faces as shown in Figure 3, while *w*_*t*_ and *w*_*b*_ stand for *w*-velocities at the top and bottom middle positions of the control volume of the continuity equation. The momentum equations (24) and (25) are written in the following forms:

(32) wi,jn+1−wi,jnΔt=pi,jn−pi+1,jnΔz+xljRin∂R∂zinpi,j+1n+pi+1,j+1n−pi,j−1n−pi+1,j−1n4Δx+conwi,jn+1Rediffwi,jn,

(33) ui,jn+1−ui,jnΔt=1Rlinpi,jn−pi,j+1nΔx+conui,jn+1Rediffui,jn,

where con*w*_*i*,*j*_^*n*^,con*u*_*i*,*j*_^*n*^,diff*w*_*i*,*j*_^*n*^ and diff*u*_*i*,*j*_^*n*^ are the finite-difference representation of convective and diffusive terms of the axial and radial momentum at the nth time level.

The Poisson equation for pressure is derived from equations (31)–(33) which takes the final forms:

(34) $$\begin{array}{c} \frac{D_{i,j}^{n+1}-D_{i,j}^{n}}{\Delta t}=A_{i,j}p_{i,j}^{n}+B_{i,j}p_{i+1,j}^{n}+C_{i,j}p_{i-1,j}^{n}+D_{i,j}p_{i,j+1}^{n}+E_{i,j}p_{i,j-1}^{n}\\ +F_{i,j}p_{i+1,j+1}^{n}+G_{i,j}p_{i+1,j-1}^{n}+H_{i,j}p_{i-1,j-1}^{n}+S_{i,j}p_{i-1,j+1}^{n}\\ +\frac{x_{li}R_{li}^{n}}{\Delta z}\left.\left\{\text{con}w_{i,j}^{n}-\text{con}w_{i-1,j}^{n}+\frac{1}{\text{Re}}\right.\left.\left[\text{diff}w_{i,j}^{n}-\text{diff}w_{i-1,j}^{n}\right.\right]\right\}\\ +\frac{1}{\Delta x}\left.\left\{x_{j}\text{con}u_{i,j}^{n}-x_{j-1}\text{con}u_{i,j-1}^{n}+\frac{x_{j}}{\text{Re}}\right.\text{diff}u_{i,j}^{n}-\frac{x_{j-1}}{\text{Re}}\text{diff}u_{i,j-1}^{n}\right\}, \end{array}$$

where *D*_*i*,*j*_^*n*+1^ represents the discretized form of the divergence of the velocity field at the (*i*, *j*) cell and the expressions for *A*_*i*,*j*_, *B*_*i*,*j*_,…, *H*_*i*,*j*_, *S*_*i*,*j*_ are the same as Mustapha et al. [15, 16]. The Poisson equation (34) for pressure is then solved using the successive overrelaxation (SOR) method to obtain the intermediate pressure field.

**Figure 4:**
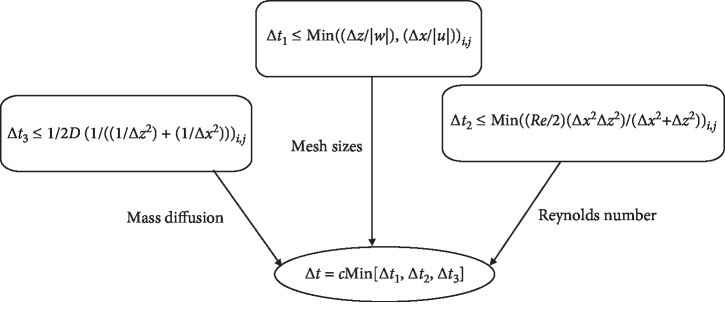
Schematic diagram for stability to obtain Δ*t*.

The increment Δ*x* is chosen to be 0.025 along *x*, while Δ*z*=0.1 along *z*.Δ*t* is chosen to be equal to or less than a prescribed stability criterion as depicted in Figure 4, where here *c* is taken to be 0.05. (cf. [25, 29]).

The number of iterations is limited up to 10. The pressure and velocities then go through a correction stage to achieve better accuracy. The process is described in [15, 16, 26, 27]. When the velocity field has been obtained, the mass concentration is calculated from the respective discretized versions of equation (26) with the relevant boundary conditions (equations (27)–(30)). The values chosen for *k*,  *α*,  Sc are 0.05, 2, and 3, respectively.

(35) $$\begin{array}{c} \frac{C_{i,j}^{n+1}-C_{i,j}^{n}}{\Delta t}=\frac{x_{lj}}{R_{li}^{n}}{\left(\frac{\partial R}{\partial t}\right)}_{li}^{n}{\left(\frac{\partial C}{\partial x}\right)}_{i,j}^{n}-\frac{u_{i,j}^{n}}{R_{li}^{n}}{\left(\frac{\partial C}{\partial x}\right)}_{i,j}^{n}-w_{i,j}^{n}\left\{{\left(\frac{\partial C}{\partial z}\right)}_{i,j}^{n}-\frac{x_{lj}}{R_{li}^{n}}{\left(\frac{\partial C}{\partial x}\right)}_{i,j}^{n}{\left(\frac{\partial R}{\partial z}\right)}_{li}^{n}\right\}\\ +\frac{1}{\text{Re}\cdot \text{Sc}}\left\{\left(\frac{1}{R_{li}^{n^{2}}}+{\left(\frac{x_{lj}}{R_{li}^{n}}{\left(\frac{\partial R}{\partial z}\right)}_{li}^{n}\right)}^{2}\right){\left(\frac{\partial^{2}C}{\partial x^{2}}\right)}_{i,j}^{n}+\left(\frac{1}{x_{lj}R_{li}^{n^{2}}}+\frac{3x_{lj}}{R_{li}^{n^{2}}}{\left(\frac{\partial R}{\partial z}\right)}_{li}^{2}-\frac{x_{lj}}{R_{li}^{n}}{\left(\frac{\partial^{2}R}{\partial z^{2}}\right)}_{li}^{n}\right){\left(\frac{\partial C}{\partial x}\right)}_{i,j}^{n}-\frac{2x_{lj}}{R_{li}^{n}}{\left(\frac{\partial R}{\partial z}\right)}_{li}^{n}{\left(\frac{\partial^{2}C}{\partial x\;\partial z}\right)}_{i,j}^{n}+{\left(\frac{\partial^{2}C}{\partial z^{2}}\right)}_{i,j}^{n}\right\}. \end{array}$$

## 4. Results and Discussions

**Figure 5:**
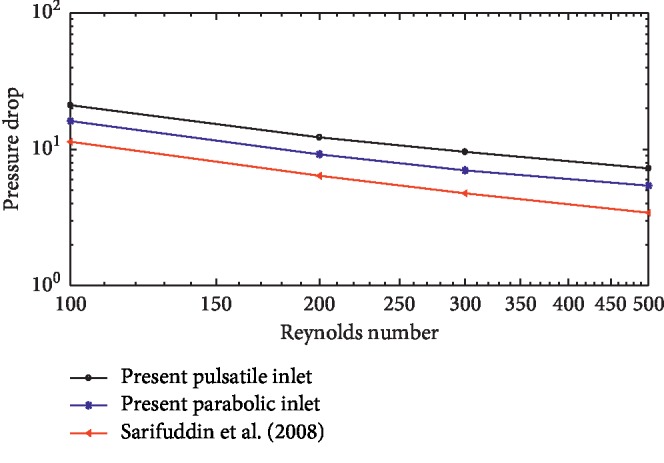
Comparison of the nondimensional pressure drop across the stenoses.

The influence of the pulsatile inlet is reflected in Figure 5 where the pressure drop in this case is higher than the one generated with the parabolic inlet. It decreases with increasing Re with a strong linear correlation between them in both cases of parabolic and pulsatile conditions (cf. Sarifuddin et al. [26]). Further, the pressure drop is seen to increase with the number of stenoses, which agrees with the experimental study of Talukder et al. [8].

**Figure 6:**
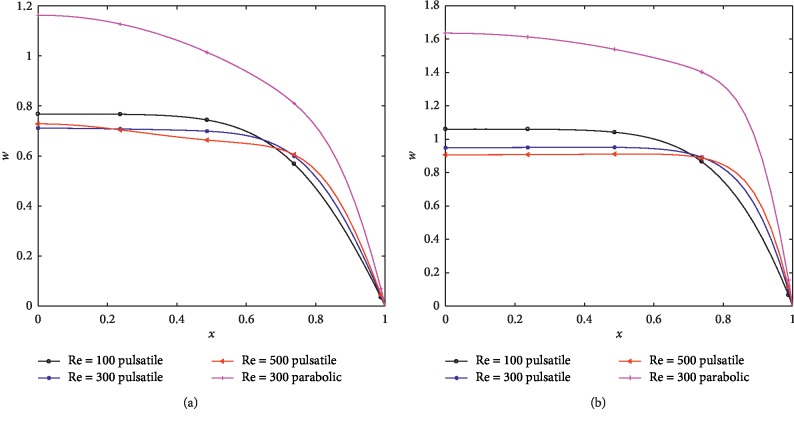
Axial velocity profiles for different Re at (*δ*_1_=0.2, *δ*_2_=0.4) (a) *z*=10. (b) *z*=19.

**Figure 7:**
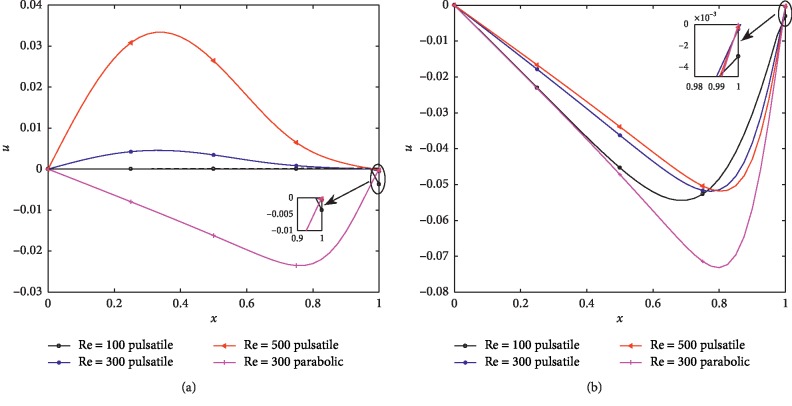
Radial velocity profiles for different Re at (*δ*_1_=0.2,  *δ*_2_=0.4) (a) *z*=10. (b) *z*=19.

The behavior of the axial and the radial velocity at the narrowest points (*z*=10 and *z*=19) for different Re are shown in Figures 6(a), 6(b), 7(a), and 7(b). The axial velocity has positive values, and it is noted that the parabolic case results in higher velocity. It is also observed that the axial velocity near the wall increases with increasing Re; however, there is a cross over at *x*=0.65 and *x*=0.73 for *z*=10 and *z*=19, respectively. Figures 7(a) and 7(b) show that the radial velocity corresponding to the pulsatile inlet assume positive values at *z*=10 except the value on the wall at (*x*=1) while negative values are observed at the narrowest point (*z*=19) and the flow velocity increases with increasing Re near the centerline while it is reduced near the wall with increasing Re. Both figures reveal that the velocities in the case of the parabolic inlet are negative and substantially less than that with the pulsatile inlet.

**Figure 8:**
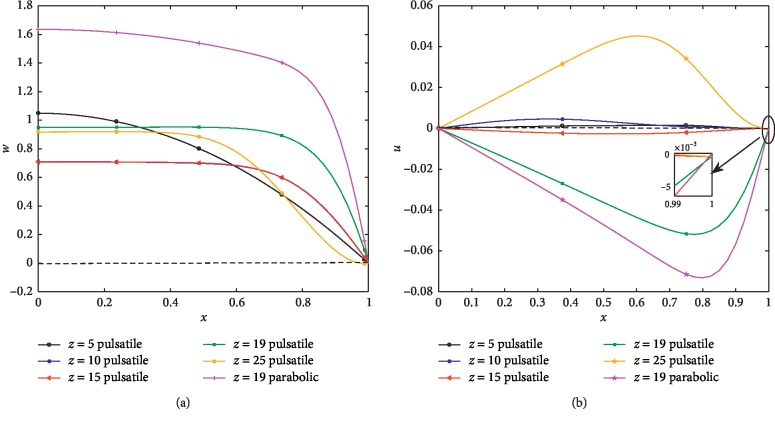
(a) Axial velocity profiles for different axial positions at Re = 300 (*δ*_1_=0.2, *δ*_2_=0.4). (b) Radial velocity profiles for different axial positions at Re = 300 (*δ*_1_=0.2, *δ*_2_=0.4).

Figure 8(a) exhibits the axial velocity profiles at different locations of the stenosed arterial segment at Re = 300 for both parabolic and pulsatile inlet conditions. At (*z*=19), the velocity in the parabolic case is higher than in the pulsatile case. A backflow occurs in the pulsatile case at the downstream of the narrowest point (*z*=19) near the wall. The curves decrease from their individual maximum at the axis as one moves away from it and finally they approach a minimum value (zero) on the wall surface. Note that the curves of the axial velocity at (*z*=10) and at (*z*=15) are coincident. The axial velocity at the critical height of the second stenosis (*z*=19) is considerably higher than that of the first stenosis (*z*=10).  Figure 8(b) shows that the radial velocity has positive values everywhere at (*z*=5), (*z*=10), and (*z*=25), excluding the position on the wall. At (*z*=15) and the narrowest point (*z*=19), the radial velocity is observed to have all negative values. The nonzero values of the radial velocity near the wall clearly reflect the influence of the radial motion of the arterial wall in the pulsatile case.

**Figure 9:**
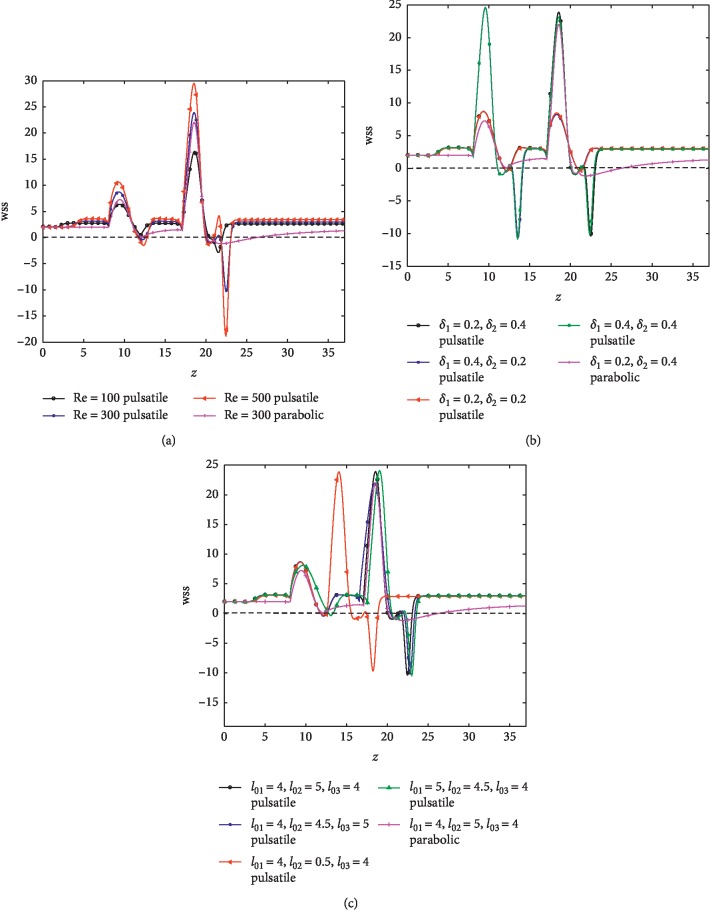
Variation of the wall shear stress along *z*. (a) For different Re (*δ*_1_=0.2,  *δ*_2_=0.4). (b) For various combinations of severities at Re = 300. (c) For various lengths and distances between stenoses at Re = 300 (*δ*_1_=0.2,  *δ*_2_=0.4).

Figure 9(a) exhibits the distribution of the wall shear stress (wss) along the arterial segment for different Re considering pulsatile as well as parabolic inlet conditions. The results show that wss for both the parabolic and pulsatile inlet conditions attain their peaks at the critical heights of the stenoses (*z*=10,19). It is observed that separation occurs (negative values of wss) only at the downstream of the second stenosis for parabolic inlet condition. In the pulsatile case, the separation zone occurs between the two stenoses with multiple separation regions at the downstream of the second stenosis. Then, wss starts to increase slowly towards the wall surface (reattachment point).

The effect of different severities on wss is depicted in Figure 9(b). In the pulsatile case, when the two stenoses have the same severities (*δ*_1_=*δ*_2_=0.2) and, (*δ*_1_=*δ*_2_=0.4), flow separation occurs at two specific places at the downstream of the first and the second stenoses with different peak values. In the case of stenoses with different severities (*δ*_1_=0.2,  *δ*_2_=0.4) and (*δ*_1_=0.4,  *δ*_2_=0.2) a larger separated region is formed at the downstream of the more severe stenoses (cf. Johnston and Kilpatrick [12]). A smaller separation region is produced in the case of pulsatile inlet condition and the peak wss is much higher than the parabolic inlet condition.

Figure 9(c) determines the effects of the length of stenoses on wss. Peak wall shear stress decreases with increasing the length of stenosis but it increases with the gap between stenosis and at this position, there is a potential that plaque would rupture whereas, at the low shear stress position, atherosclerotic development may be induced. These phenomena of separation and reattachment are due to the adverse pressure in these regions and are believed to be responsible for the malfunctioning of the cardiovascular system having atherosclerotic plaque.

**Figure 10:**
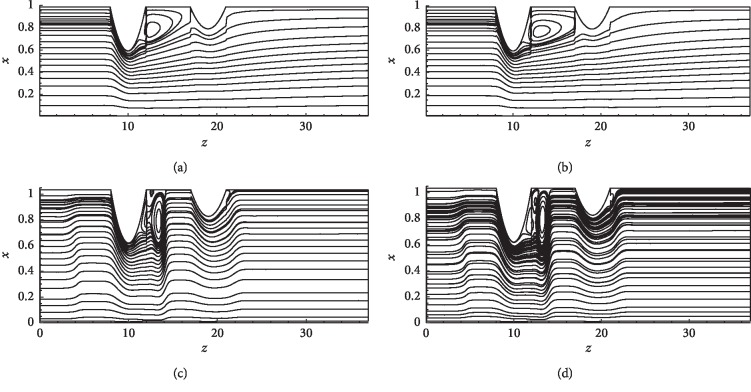
Pattern of streamlines for (*δ*_1_=0.4,  *δ*_2_=0.2). (a) Re = 300 parabolic inlet. (b) Re = 500 parabolic inlet. (c) Re = 300 pulsatile inlet. (d) Re = 500 pulsatile inlet.

Figures 10(a)–10(d) show the instantaneous patterns of streamlines governing the flow of blood through the stenoses in case of (*δ*_1_=0.4,  *δ*_2_=0.2) for both parabolic and pulsatile inlet conditions at Re = 300 and Re = 500. In the parabolic case, only one recirculation zone developed between the two stenoses where separation occurs at *z*=11 (c.f Figure 9(b)). In the case of the pulsatile inlet, a multiple recirculation region is noted in between the two stenoses (separation point *z*=11). Thus, an increase in Re and a consideration of the pulsatile flow increase the number of the recirculation region.

**Figure 11:**
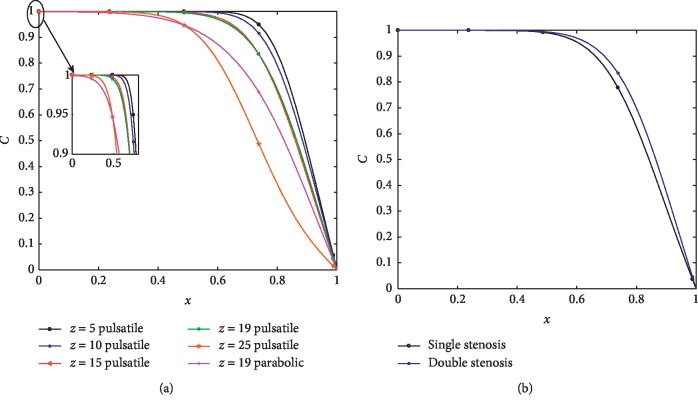
(a) Variation of mass concentration profiles for different axial positions at Re = 300. (b) Mass concentration profiles of double and single stenoses at *t* = 30.

Figure 11(a) exhibits the profiles of mass concentration at different positions for both pulsatile and parabolic inlet conditions at Re = 300. The mass concentration at each axial position converges to zero according to the wall condition. The mass concentration gets distorted at the downstream of the stenoses which could be due to the separation of flow at the downstream of the stenoses. Along the upstream of stenoses, the flow velocity and wall shear stress (Figure 8(a)) increase as the flow gets accelerated towards the throat leading to the increase of solute concentration. It is also observed that the concentration at the throat (*z*=19) is much higher for pulsatile flow than the parabolic one.  Figure 11(b) exhibits the evolution of mass concentration at the throat of stenosis corresponding to single and double stenosis for pulsatile inlet condition. The mass concentration is higher at the throat in the case of double stenosis compared to the single one.

**Figure 12:**
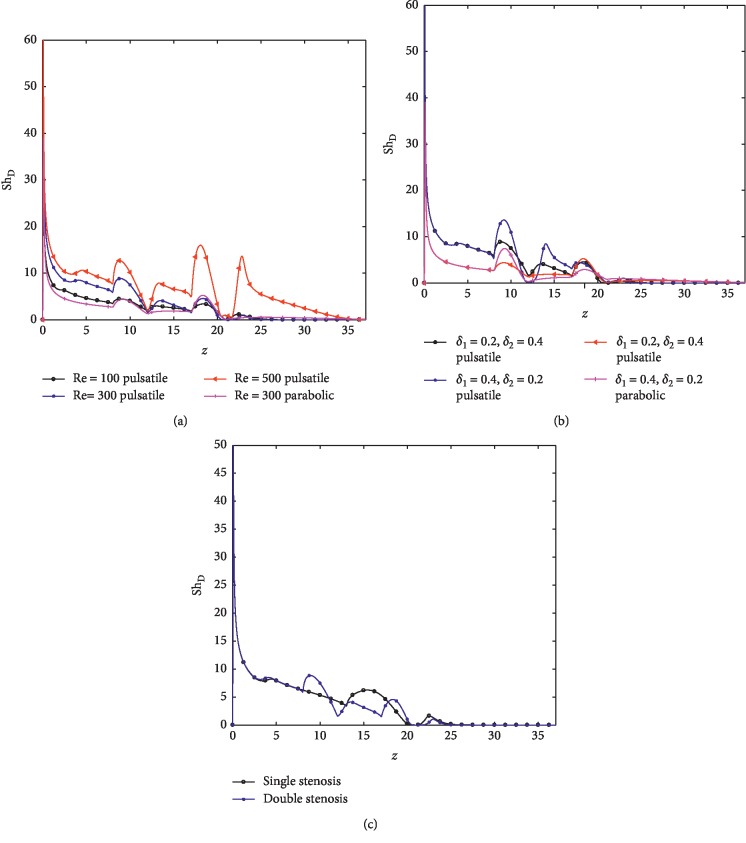
Distribution of local Sherwood number for (a) different Reynolds numbers, (b) different severity of stenoses, and (c) double and single stenoses.

The mass flux to the arterial wall which is quantified through the Sherwood number over the entire stenosed arterial segment is examined and exhibited in Figures 12(a)–12(c). The Sherwood number defined by Sh_*D*_=2*R*_0_*c*_*l*_/*D*_m_Δ*C* where *c*_*l*_ is the local mass flux to the arterial wall and 2*R*_0_ is the inlet diameter of the artery. It is observed that Sh_*D*_ increases with increasing Re while Sh_*D*_ distribution appreciably changes specifically at the throat, between the two stenoses and at the downstream position. The highest mass transfer is experienced at the upstream, while the minimum value occurs at the downstream of the stenoses. Note that pulsatile flow increases the Sherwood number and that it is much higher in the case of double stenosis.

**Figure 13:**
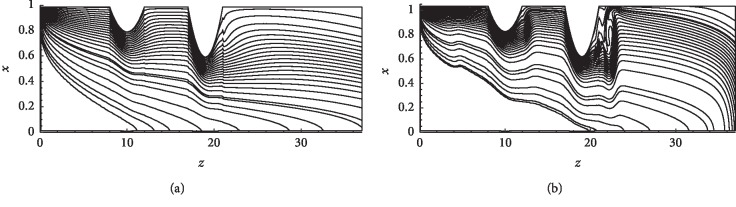
Iso-concentration lines with different inlet conditions. (a) Parabolic inlet. (b) Pulsatile inlet.

The iso-concentration lines considering pulsatile as well as parabolic inlet conditions based on (*δ*_1_=0.2,  *δ*_2_=0.4) are displayed in Figures 13(a) and 13(b). The iso-concentration lines for parabolic and pulsatile inlets have different distributions with multiple recirculation regions nearby the downstream of the more severe stenosis in the pulsatile case. The general trend of the iso-concentration lines is that they move away from the inlet region towards the upstream of the stenosis and correspondingly impair the mass transport in this region, while they adhere to the outline of the stenosis at both the upstream and downstream ends. At this region of low wss (compare with Figure 9), cholesterol may tend to accumulate and cause more severe stenosis. This observation conforms with Schneiderman et al. [30].

## 5. Conclusion

The hemodynamics of the pulsatile flow and the transport of mass in an arterial segment having a couple of stenoses have been studied in relation to the distensibility of the vessel wall. Predicted results show that the pulsatile inlet and double stenoses with varying severity affect the flow characteristics significantly, especially the development of the recirculation zone and the peak value of the wall shear stress. It is also predicted that the concentration at the throat (*z* = 19) is much higher for pulsatile flow than the parabolic inlet condition. Moreover, the pair of stenoses contributes much to the mass concentration than the case of single stenosis. The mass flux to the arterial wall (Sherwood number) does increase with the increasing values of Re and here too, mass flux increases with the flow pulsatility and the presence of double stenoses. At the downstream, cholesterol may tend to accumulate and causes more severe stenosis. For severe stenoses, the peak value of the wall shear stress is higher in the pulsatile flow case and the iso-concentration lines show more recirculation regions nearby the downstream end and their lengths are longer. In conclusion, the results presented agree well with physical observations and provide an insight into the link between atherosclerosis, stenosis, and the pattern of mass transport.

Though the detailed knowledge of the dynamical variables is possible and provides useful elements, the mechanism of influence of the haemodynamical factors in the arterial disease is not clear. The characteristics of the red cells must be taken into consideration by including a shear-dependent viscosity in the diffusion terms in time-dependent flows highlighting the scope of further work. All these mechanical and biochemical aspects related to the biofluid dynamics are of some importance and demand further investigation. A great deal of work is needed to establish the rheological parameters for the physiological values and to understand the connection of the issues with biological facts.

## Data Availability

The data on blood flow parameters used for analysis and validation purposes are from previously reported studies and datasets, which have been cited.
